# Machine Learning Screens Potential Drugs Targeting a Prognostic Gene Signature Associated With Proliferation in Hepatocellular Carcinoma

**DOI:** 10.3389/fgene.2022.900380

**Published:** 2022-06-28

**Authors:** Jun Liu, Jianjun Lu, Wenli Li, Wenjie Mao, Yamin Lu

**Affiliations:** ^1^ Department of Clinical Laboratory, Yue Bei People’s Hospital, Shantou University Medical College, Shaoguan, China; ^2^ Medical Research Center, Yue Bei People’s Hospital, Shantou University Medical College, Shaoguan, China; ^3^ Department of Medical Affairs, First Affiliated Hospital of Sun Yat-sen University, Guangzhou, China; ^4^ Reproductive Medicine Center, Yue Bei People’s Hospital, Shantou University Medical College, Shaoguan, China; ^5^ Emergency Department, Yue Bei People’s Hospital, Shantou University Medical College, Shaoguan, China

**Keywords:** hepatocellular carcinoma, proliferation, gene signature, immune checkpoint inhibitors, antitumor drugs

## Abstract

**Background:** This study aimed to screen potential drugs targeting a new prognostic gene signature associated with proliferation in hepatocellular carcinoma (HCC).

**Methods:** CRISPR Library and TCGA datasets were used to explore differentially expressed genes (DEGs) related to the proliferation of HCC cells. Differential gene expression analysis, univariate COX regression analysis, random forest algorithm and multiple combinatorial screening were used to construct a prognostic gene signature. Then the predictive power of the gene signature was validated in the TCGA and ICGC datasets. Furthermore, potential drugs targeting this gene signature were screened.

**Results:** A total of 640 DEGs related to HCC proliferation were identified. Using univariate Cox analysis and random forest algorithm, 10 hub genes were screened. Subsequently, using multiplex combinatorial screening, five hub genes (FARSB, NOP58, CCT4, DHX37 and YARS) were identified. Taking the median risk score as a cutoff value, HCC patients were divided into high- and low-risk groups. Kaplan-Meier analysis performed in the training set showed that the overall survival of the high-risk group was worse than that of the low-risk group (*p* < 0.001). The ROC curve showed a good predictive efficiency of the risk score (AUC > 0.699). The risk score was related to gene mutation, cancer cell stemness and immune function changes. Prediction of immunotherapy suggetsted the IC50s of immune checkpoint inhibitors including A-443654, ABT-888, AG-014699, ATRA, AUY-922, and AZ-628 in the high-risk group were lower than those in the low-risk group, while the IC50s of AMG-706, A-770041, AICAR, AKT inhibitor VIII, Axitinib, and AZD-0530 in the high-risk group were higher than those in the low-risk group. Drug sensitivity analysis indicated that FARSB was positively correlated with Hydroxyurea, Vorinostat, Nelarabine, and Lomustine, while negatively correlated with JNJ-42756493. DHX37 was positively correlated with Raltitrexed, Cytarabine, Cisplatin, Tiotepa, and Triethylene Melamine. YARS was positively correlated with Axitinib, Fluphenazine and Megestrol acetate. NOP58 was positively correlated with Vorinostat and 6-thioguanine. CCT4 was positively correlated with Nerabine.

**Conclusion:** The five-gene signature associated with proliferation can be used for survival prediction and risk stratification for HCC patients. Potential drugs targeting this gene signature deserve further attention in the treatment of HCC.

## Introduction

As one of the most common cancers worldwide, hepatocellular carcinoma (HCC) is currently the third leading cause of cancer-related death (Cronin et al., 2018). In the past few decades, the effects of drug resistance and long-term toxicity of systemic therapy on overall survival (OS) have limited its application, making systemic therapy only used for advanced HCC. Before 2017, the anti-angiogenic tyrosine kinase inhibitor sorafenib was almost the only option for systemic treatment for advanced HCC patients. Subsequently, several molecularly targeted therapeutic agents, including lenvatinib, regorafenib, and ramucirumab, have broadened the treatment options for advanced HCC. In recent years, the important role of immune system regulation in HCC has made immunotherapy the focus of HCC research efforts.

Immune checkpoint inhibitors (ICIs) are monoclonal antibodies that block the interaction of checkpoint proteins with their ligands, thereby preventing T cell inactivation. The antitumor effects of immunotherapy drugs are based on immune checkpoint-mediated inhibition of programmed cell death-1 (PD-1), programmed cell death ligand 1 (PD-L1), and cytotoxic T lymphocyte-associated protein 4 (CTLA-4). Previous studies have shown that immune checkpoint inhibitors, including anti-PD-1, anti-PD-L1, and anti-CTLA-4 antibodies, have shown potential therapeutic promise for advanced HCC (Zongyi and Xiaowu, 2020). The combination of the anti-PDL1 antibody atezolizumab and the vascular endothelial growth factor-neutralizing antibody avastin is about to become the standard treatment for HCC. Compared with sorafenib, the immunotherapy combination regimen based on atezolizumab and avastin showed a clear advantage in improving the survival rate of patients with unresectable HCC. In addition, the anti-PD1 drugs nivolumab and pembrolizumab began to be used after the use of anti-angiogenic tyrosine kinase inhibitors. Currently, the combination of HCC checkpoint immunotherapy with other systemic or local treatments is considered the most promising treatment option for HCC. And immunotherapy is expected to be integrated into early and mid-stage treatment regimens.

However, on the one hand, the severe toxicity of systemic drugs has slowed the development of new HCC drugs over the past decade (Busato et al., 2019). On the other hand, the predictive power and accuracy of traditional pathological staging have been shown to be insufficient due to the marked heterogeneity of HCC. The lack of predictive biomarkers makes the choice of immunotherapy over kinase inhibitors an empirical treatment decision that balances antitumor efficacy and drug toxicity (Fulgenzi et al., 2021). The identification and validation of predictive biomarkers and the screening of more effective immunotherapeutic drugs or drug combinations are urgently needed for HCC immunotherapy (Sangro et al., 2021).

As we know, HCC cells are characterized by fast growth and strong invasiveness. Therefore, proliferation-related gene signatures are potential prognostic biomarkers for HCC. Previous researches suggest that DEPDC1 can promote the occurrence and proliferation of HCC (Qu et al., 2019). High expression of E2F1 can promote cancer cell proliferation by activating PKC-α phosphorylation in HCC (Lin et al., 2019). YTHDF2 can inhibit the proliferation of cancer cells by destroying the stability of EGFR mRNA in HCC (Zhong L. et al., 2019). In addition, in terms of microRNA, miR-424-5p can inhibit the proliferation and invasion of HCC cells by targeting TRIM29 (Du et al., 2019). MiR-125a-5p can inhibit the growth and metastasis of liver cancer cells by targeting TRIAP1 and BCL2L2 (Ming et al., 2019). MiR-490-5p inhibits the proliferation, migration and invasion of cancer cells by directly regulating ROBO1 in HCC (Chen et al., 2019). MiRNA-217 can inhibit the proliferation of cancer cells by regulating KLF5 in HCC (Gao et al., 2019). MiR-664 may target SIVA1 to promote proliferation, migration and invasion in HCC (Wang X. et al., 2019). In terms of long non-coding RNAs (lncRNAs), LncRNAs A1BG-AS1 can inhibit the proliferation and invasion of HCC cells by targeting miR-216a-5p (Bai et al., 2019). While LncRNA 01123, LncRNA HAGLROS, LncRNA MNX1-AS1, LncRNA CRNDE, and LncRNA RNA CCAT2 can promote the proliferation and metastasis of HCC cells (Ji et al., 2019a; Ji et al., 2019b; Liu et al., 2019; Wei H. et al., 2019; Xiao et al., 2020). Therefore, the above genes have potential value as prognostic biomarkers in HCC.

In this study, we used the CRISPR Library and the Cancer Genome Atlas (TCGA) database to screen for important genes related to the proliferation of HCC cells. Then, hub genes most relevant to the prognosis of HCC patients were identified and used to establish a gene signature for survival prediction. Subsequently, the prognostic values of the gene signature were confirmed both in the training set and validation set. Time-dependent receiver operating characteristic (t-ROC) curve was used to verify the prediction accuracy of the survival model. Associations of risk scores with genetic mutations, cancer cell stemness and immune function were analyzed, respectively. Finally, drugs targeting this proliferation-related gene signature were identified. In conclusion, this study comprehensively analyzed the prognostic value of a new proliferation-related gene signature in HCC. This gene signature can not only be used for prognostic assessment and risk stratification of HCC patients, but also is expected to be a therapeutic target for HCC. Furthermore, therapeutic drugs targeting this gene signature may have potential therapeutic prospects.

## Materials and Methods

### Data Source and Identification of Proliferation-Related Differentially Expressed Genes

The RNASeq data and clinical information used to construct the prognostic gene signature were downloaded from the TCGA HCC dataset (*n* = 365). The RNASeq data and clinical information used to verify the gene signature were downloaded from the International Cancer Genome Consortium (ICGC) HCC dataset (*n* = 232). The limma package was used to perform differentially expressed gene analysis between tumor and matched normal tissues. Candidates with false discovery rate (FDR) <0.05 and multiple of change >1 were considered to be significantly upregulated in tumor tissues. The genome-wide CRISPR screening of HCC cells was downloaded from the DepMap portal (https://depmap.org/portal/download/). The CERES algorithm was used to calculate the dependency scores of candidate genes (Meyers et al., 2017). Candidate genes were defined as proliferation-related genes. The above three databases are public. Therefore, this study did not require the approval of the local ethics committee.

### Candidate Gene Selection and Gene Signature Establishment

Random forest is a machine learning algorithm based on decision tree, which is a nonlinear classifier and can be used for sample classification or regression tasks. The method of random forest to evaluate the importance of features is to calculate how much each feature contributes to different decision trees in random forest, then take the average value, and compare the contribution of different features. In this study, using univariate Cox regression with a *p* value < 0.01, the candidate genes that are most relevant to the prognosis of HCC patients were identified. Next, we used random forest to rank the importance of genes and selected the top 10 hub genes. Subsequently, we identified a gene signature with a smaller number of genes and a more significant *p* value from multiple combinations of 10 hub genes to construct a survival model. The single-sample gene set enrichment analysis (ssGSEA) algorithm was used to quantify the performance of proliferation-related pathways and transcription factors. In addition, gene mutations, cancer cell stemness and immune function changes can affect tumor proliferation and the prognosis of HCC, so we explored the correlations between the gene signature and gene mutations/mRNSsi/immune functions.

### Survival Analysis Based on Risk Score

Taking the median risk score as the cut-off value, we divided HCC patients into high- and low-risk groups. Then the prognosis of the two groups was compared in the training set and the validation set, respectively. Kaplan-Meier method was used for survival analysis. ROC curve was used to evaluate the predictive accuracy of the risk score. And t-ROC was used to evaluate the predictive ability (R package “survival-ROC”) (Heagerty et al., 2000). Cox proportional hazard regression model was used to evaluate the importance of each parameter to OS. In addition, a two-factor survival analysis combining risk score and proliferation-related pathways was also performed to evaluate the impact of risk score and proliferation-related pathways on the prognosis of HCC patients.

### Establishment and Evaluation of Nomogram for Predicting OS of HCC Patients

Nomogram is an effective tool for predicting the prognosis of cancer patients by simplifying complex statistical prediction models into maps that assess the probability of individual patients’ OS (Park, 2018). In this study, we constructed a nomogram based on the five-gene signature to evaluate the probability of OS in HCC patients at 1-, 3-, and 5-year. Meanwhile, the predicted probability of the nomogram was compared with the measured probability by the calibration curve to verify the accuracy of the nomogram. In addition, t-ROC curve was used to evaluate the survival prediction ability of the nomogram. Decision curve analysis (DCA) curve was used to evaluate the clinical benefit of the nomogram.

### Drug Discovery Based on Risk Score

In order to find candidate drugs that show potential efficacy in the high-risk group, we used the half-maximum inhibitory concentration (IC50) of each HCC patient to evaluate their treatment response on Genomics of Drug Sensitivity in Cancer (GDSC) (https://www.cancerrxgene.org/) (Geeleher et al., 2014).

### Drug Sensitivity Analysis of Five Hub Genes

The drug sensitivity data was downloaded from the CellMiner™ database (version: 2020.3, database: 2.4.2, https://discover.nci.nih.gov/cellminer/home.do) (Reinhold et al., 2012). The R packages “impute,” “limma,” “ggplot2,” and “ggpubr” were used for data processing and visualization.

### Bioinformatics and Statistical Analysis

IBM SPSS Statistics 20 (IBM Corp., Armonk, NY, United States) and R software (version 3.5.2, https://www.r-project.org) were used to analyze data and draw graphs. Z-score were used to normalize the ssGSEA score. Principal component analysis was conducted by using the Rtsne R package. The log-rank test was used to assess the differences. The “wilcox.test” function was used to compare the risk scores between groups.

## Results

### Schematic Diagram of Research Design

**FIGURE 1:**
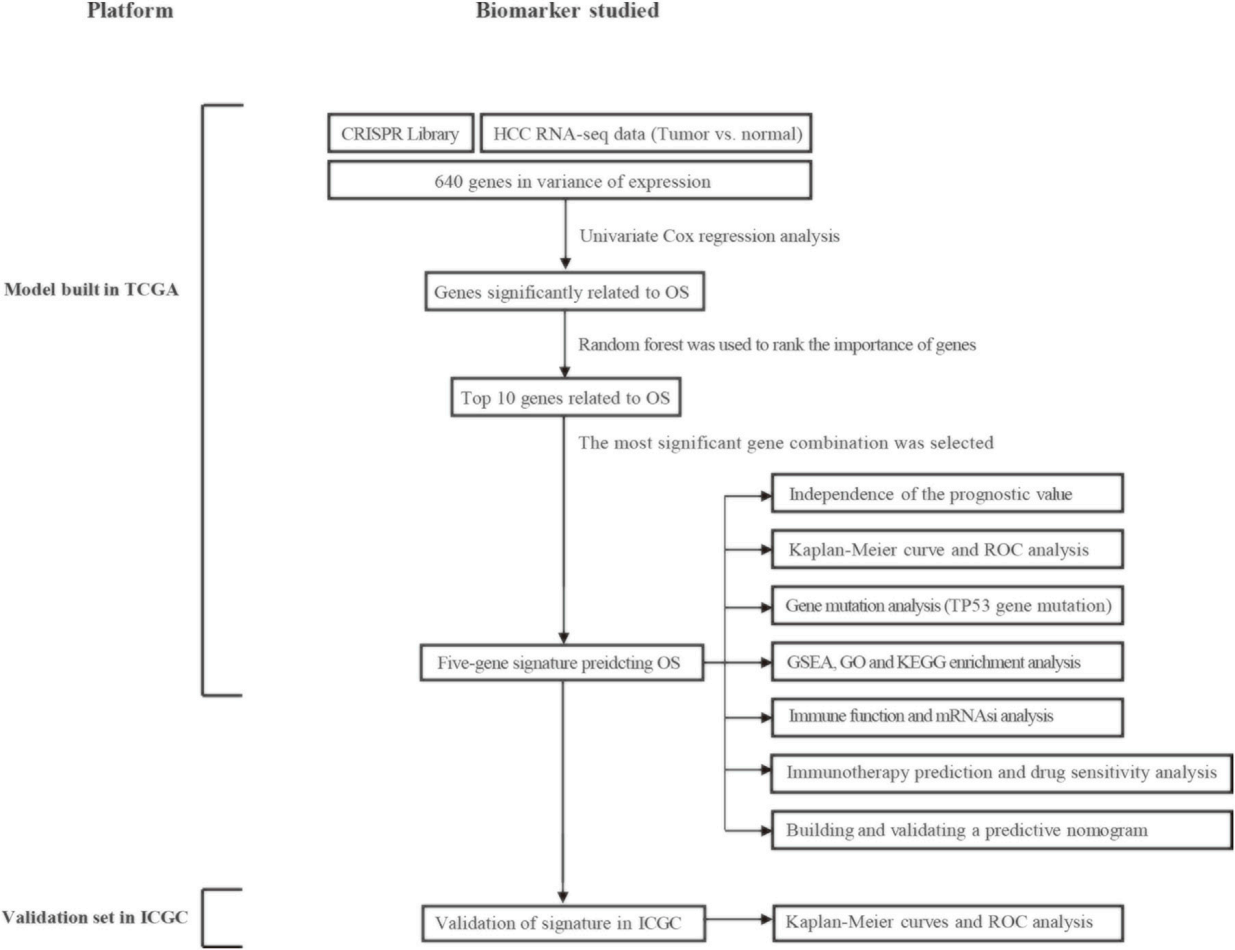
Overall flowchart of this study. HCC, hepatocellular carcinoma; OS, overall survival; ROC, receiver operating characteristic; GSEA, gene set enrichment analysis; GO, gene ontology; KEGG, kyoto encyclopedia of genes and genomes; mRNAsi, mRNA expression-based stemness index.

Figure 1 shows the entire workflow of this research. Firstly, using the CRISPR Library and TCGA HCC dataset, differentially expressed genes (DEGs) related to HCC proliferation were screened out. Then, univariate Cox regression analysis was used to screen promising candidates. Next, the random forest algorithm and multiple combinatorial screening methods were used to establish a prognostic gene signature. Specifically, we screened genes associated with overall survival in HCC by univariate COX regression, and then used random forests to rank the importance of these survival-related genes and listed the top 10 genes. We then randomly combined these 10 genes and constructed a risk model by multivariate COX regression. Subsequently, we calculated and ranked the *p*-values for each model by K-M survival analysis. Furthermore, we screened out the risk model with the smallest *p* value and the relatively small number of genes. Finally, the prognostic values of the gene signature were evaluated in the training set and validation set, respectively.

### Establishment of Proliferation-Related Prognostic Gene Signature

**FIGURE 2:**
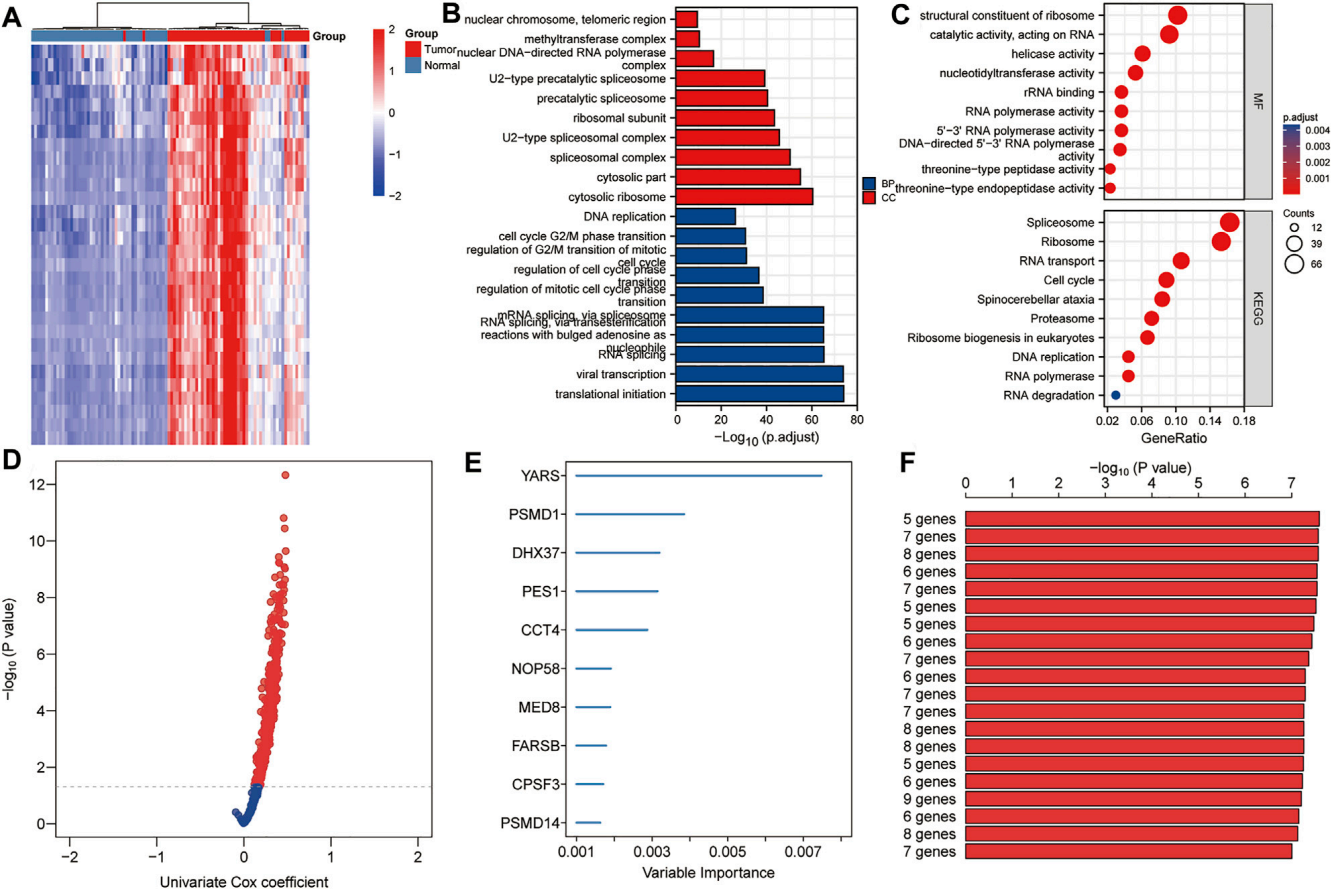
Establishment of prognostic gene markers related to cancer cell proliferation in HCC. **(A)** Heat map showing significantly DEGs in HCC related to cancer cell proliferation. Using the RNA sequencing data of the TCGA HCC cohort and the CRISPR Library, 640 DEGs related to proliferation in HCC were screened out (|log2FC| > 1 and FDR < 0.05). **(B,C)** GO and KEGG analysis revealed important biological processes, cell components and KEGG pathways enriched by 640 DEGs. **(D)** Using univariate Cox analysis, candidates related to prognosis were identified (*p* < 0.05). **(E)** Using random forest, the top 10 most characteristic genes are screened out. **(F)** A combination with a relatively small number of genes and a relatively significant *p* value was selected to construct a survival prediction model from a variety of combinations of 10 genes. DEGs, differentially expressed genes; FDR, false discovery rate; BP, biological processes; CC, cell components; MF, molecular function.

A total of 640 DEGs in HCC were identified, with |log2FC| > 1 and FDR < 0.05 as the thresholds. The heat map shows the expression profiles of some DEGs related to proliferation in HCC (Figure 2A). As shown in Figures 2B,C, biological processes significantly enriched by 640 DEGs included ribosomal subunit, U2-type spliceosomal complex, spliceosomal complex, cytosolic part and cytosolic ribosome; Significantly enriched cell components included mRNA splicing, *via* spliceosome, RNA splicing *via* transesterification reactions with bulged adenosine as nucleophile, RNA splicing, viral transcription, and translational initiation; Significantly enriched molecular function included structural constituent of ribosome, catalytic activity acting on RNA, helicase activity, nucleotidyltransferase activity and rRNA binding. In addition, significantly enriched pathways included spliceosome, ribosome, RNA transport, cell cycle and spinocerebellar ataxia. Using *p* < 0.01 as the threshold for univariate Cox regression, candidate genes related to the prognosis of HCC patients were identified (Figure 2D). Subsequently, we used random forest ranking to rank candidate genes and screened out the top ten relatively important genes (Figure 2E). Next, we selected a gene combination with a smaller number of genes and a more significant *p* value from multiple combinations of ten hub genes to construct a survival prediction model (Figure 2F). Finally, five hub genes were used to construct a prognostic model of HCC: risk score = 0.010 * FARSB + 0.07 * NOP58 + 0.001 * CCT4 − 0.026 * DHX37 + 0.022 * YARS.

### Risk Score Based on the Five-Gene Signature Was an Independent Prognostic Factor for HCC

**FIGURE 3:**
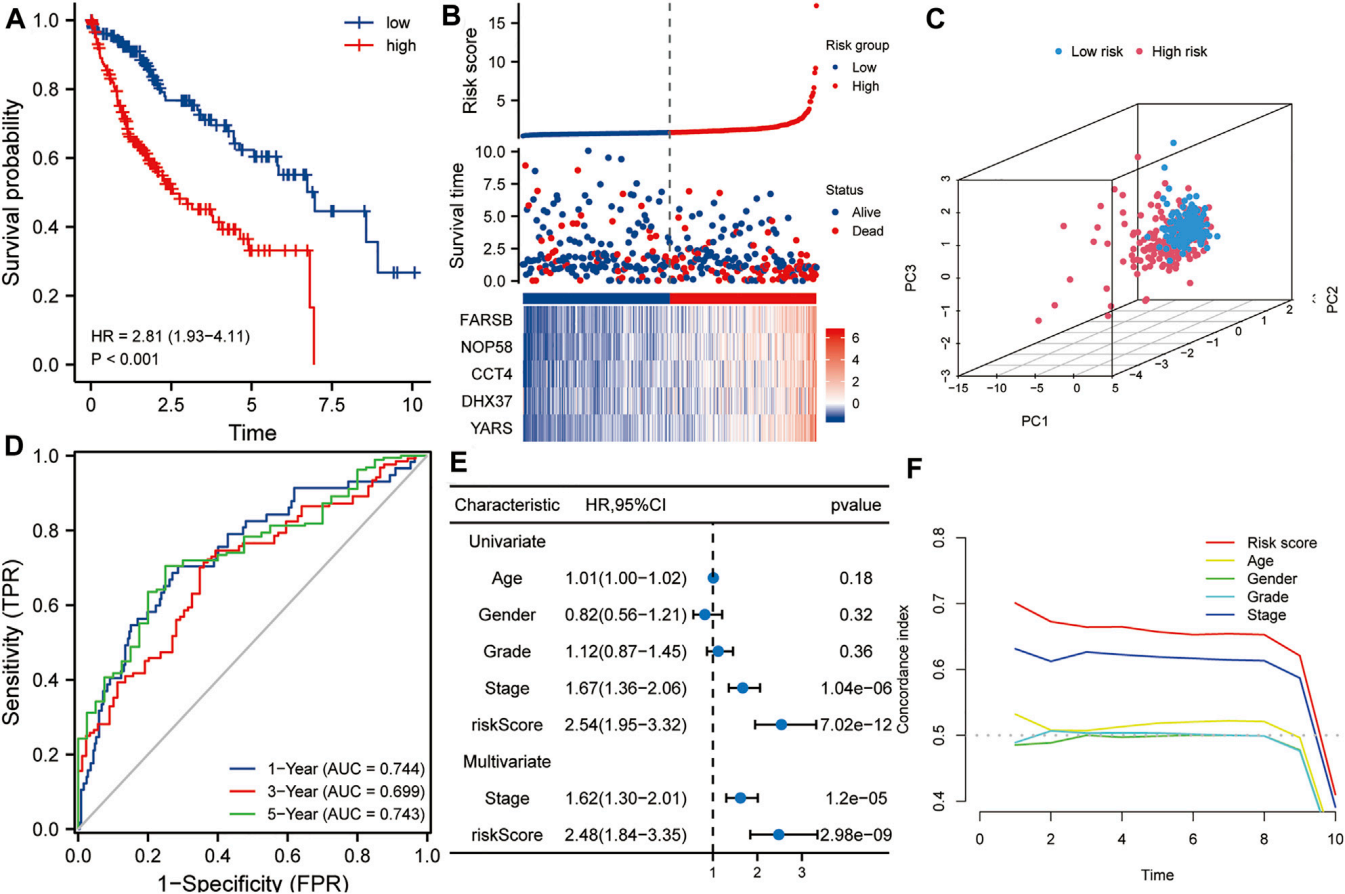
The risk score predicts poor survival in the training set. **(A)** Kaplan-Meier analysis showed that HCC patients with higher risk scores had a worse overall survival rate. **(B)** The risk score distribution, survival profile and heat map of patients in the high- and low-risk groups in the training set. **(C)** Principal component analysis suggested that risk score could be used as a new dimension to evaluate the prognosis of HCC patients. **(D)** The ROC curve showed the prediction efficiency of the risk score in the training set (AUC > 0.699). **(E)** Univariate and multivariate Cox regression analysis showed that risk score was an independent risk factor for OS in HCC patients. **(F)** The tROC analysis showed that the predictive power of risk score was significantly higher than that of other clinical characters. HR, hazard ratio; OS, overall survival; tROC, time-dependent receiver operating characteristics.

The TCGA HCC dataset was used as a training set to evaluate the prognostic values of this five-gene signature. As shown in Figure 3A, Kaplan-Meier analysis showed that the prognosis of the high-risk score group was worse than that of the low-risk score group (*p* < 0.001). The high- and low-risk score groups were defined by risk scores based on the five-gene signature. The median risk score calculated from the risk model was 0.867. Taking the median risk score of HCC patients as a cutoff value, we divided HCC patients into high-risk and low-risk groups. Patients with a risk score higher than 0.867 were classified as high-risk group, while those with a risk score lower than 0.867 were classified as low-risk group. Subsequently, in order to evaluate the relationship between the five-gene signature and the prognosis of HCC patients, we took the median of the risk scores of 338 HCC patients from the training set as the cut-off value, divided these patients into high- and low-risk groups, and compared the survival status and the expressions of the five hub genes between the two groups. The results showed that the prognosis of the high-risk group was worse than that of the low-risk group, and the expression levels of five hub genes in the high-risk group were higher than that of the low-risk group (Figure 3B). Next, Principal component analysis suggested that risk score could be used as a new dimension to assess the prognosis of HCC patients (Figure 3C). The ROC curve showed that the AUCs of the risk score for predicting 1-year, 3-year, and 5-year survival rates were 0.744, 0.699, and 0.743, respectively, indicating that the risk score was a good model for predicting the survival rate of HCC patients (Figure 3D). Univariate and multivariate Cox regression analysis showed that risk score based on five-gene signature (HR = 2.48, *p* < 0.001) and pathological stage (HR = 1.62, *p* < 0.001) were independent risk factors affecting OS in HCC patients (Figure 3E). Besides, tROC analysis showed that the survival predictive ability of risk score was significantly higher than other clinicopathological characteristics (Figure 3F).

### Verifying the Prognostic Values of the Five-Gene Signature in the Validation Set

**FIGURE 4:**
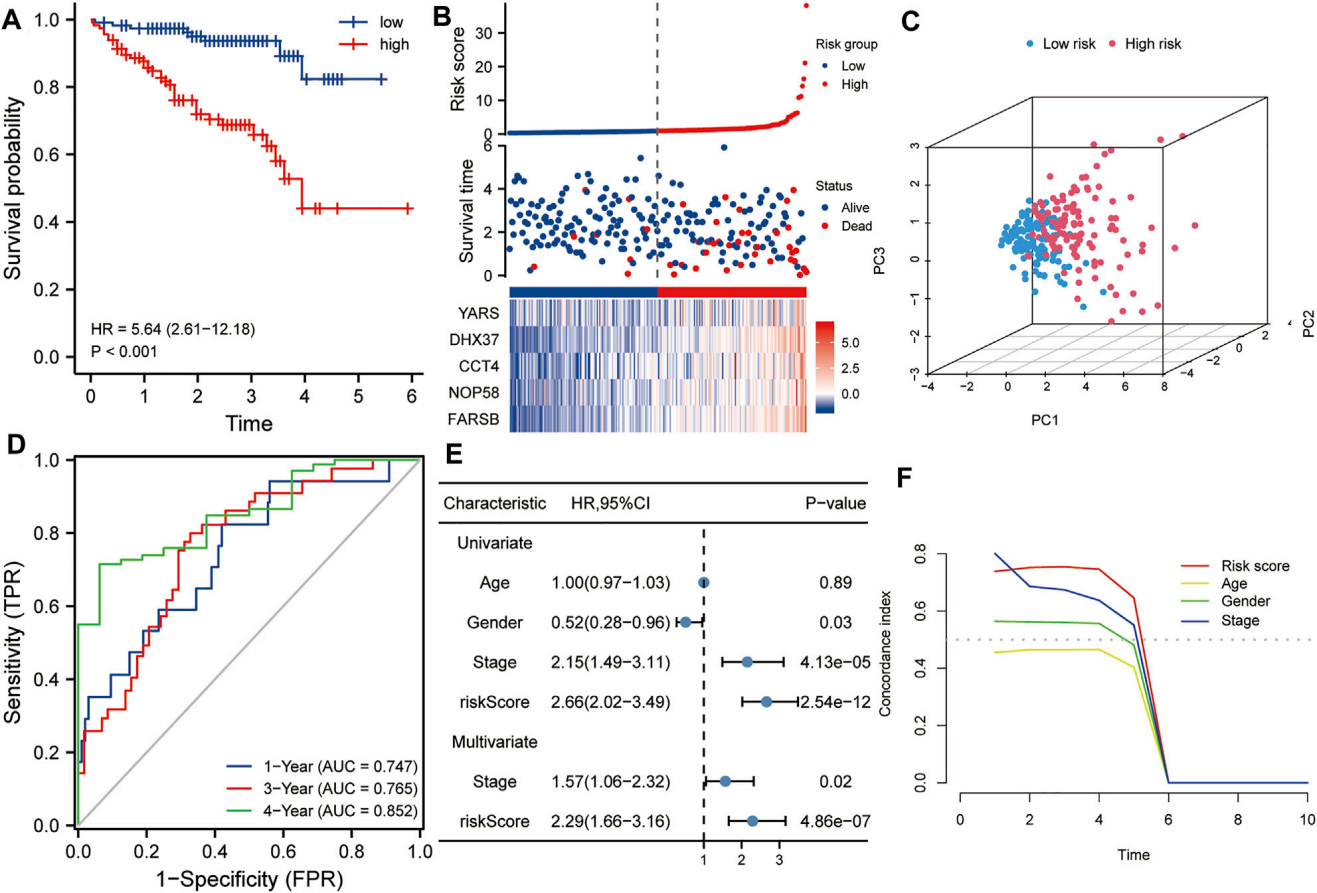
Validation of the risk score in the ICGC dataset. **(A)** Kaplan-Meier analysis showed that HCC patients with higher risk scores had a worse overall survival rate. **(B)** The risk score distribution, survival profile and heat map of patients in the high- and low-risk groups in the ICGC dataset. **(C)** Principal component analysis suggested that the risk score in the ICGC data set was a new dimension for evaluating the prognosis of patients. **(D)** The ROC curve shows the survival prediction efficiency of the risk score in the ICGC data set (AUC > 0.74). **(E)** Univariate and multivariate Cox regression analysis showed that risk score is an independent risk factor for OS in HCC patients. **(F)** tROC analysis showed that the predictive power of risk score was significantly higher than other clinical characters. HR, hazard ratio; OS, overall survival; tROC, time-dependent receiver operating characteristics.

The ICGC HCC dataset was used as a validation set to verify the robustness of this five-gene signature. Kaplan-Meier analysis showed that the prognosis of the high-risk group was worse than that of the low-risk group (*p* < 0.001, Figure 4A). Similarly, taking the median of the risk scores of 232 HCC patients from the validation set as the cutoff value, we divided these patients into high- and low-risk groups, and compared the survival status and the expression levels of five hub genes between the two groups. The results showed that the prognosis of the high-risk group was worse than that of the low-risk group, and the expression levels of the five hub genes in the high-risk group were higher than that of the low-risk group (Figure 4B). Principal component analysis also suggested that risk score could be used as a new dimension to assess the prognosis of HCC (Figure 4C). The ROC curve showed that the AUCs of the risk score for predicting 1-year, 3-year, and 5-year survival rates were 0.747, 0765, and 0.852, respectively, which further indicated that the risk score was a good model for predicting the survival rate of HCC patients (Figure 4D). Univariate and multivariate Cox regression analysis showed that risk score (HR = 2.29, *p* < 0.001) and pathological stage (HR = 1.57, *p* < 0.05) were independent risk factors affecting OS in HCC patients (Figure 4E). In addition, tROC analysis showed that the survival predictive ability of risk score was significantly higher than that of other clinicopathological characteristics (Figure 4F).

### Correlations Between Risk Score and Proliferation-Related Pathways and Corresponding Two-Factor Survival Analysis

**FIGURE 5:**
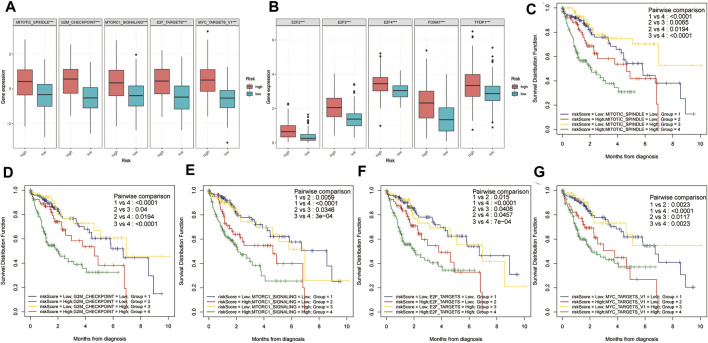
Two-factor survival analysis combining proliferation-related pathways and risk scores. **(A)** The proliferation-related pathways Z-scores in the high-risk group were significantly higher than those in the low-risk group. **(B)** The proliferation-related transcription factor Z-scores of high-risk patients were significantly higher than those of low-risk patients. **(C–G)** Two-factor survival analysis combining risk score and proliferation-related pathway Z-scores showed that high-risk score and high proliferation-related pathway Z-Scores predicted the worst prognosis.

Using the ssGSEA algorithm, the Z-scores of some proliferation-related pathways and some proliferation-related transcription factors were calculated. Subsequently, the Z-scores of proliferation-related pathways and the Z-scores of proliferation-related transcription factors between the high and low-risk groups were compared, respectively. As shown in Figure 5A, the Z-scores of the proliferation-related pathways in the high-risk group were higher than those in the low-risk group. Meanwhile, as shown in Figure 5B, the Z-scores of the proliferation-related transcription factor of the high-risk group were higher than those of the low-risk group. Subsequently, a two-factor survival analysis combining risk score and proliferation-related pathway Z-scores showed that high risk score and high proliferation-related pathway Z-scores predicted the worst prognosis (Figures 5C–G).

### Differences in Gene Mutations Between High- and Low-Risk Groups

**FIGURE 6:**
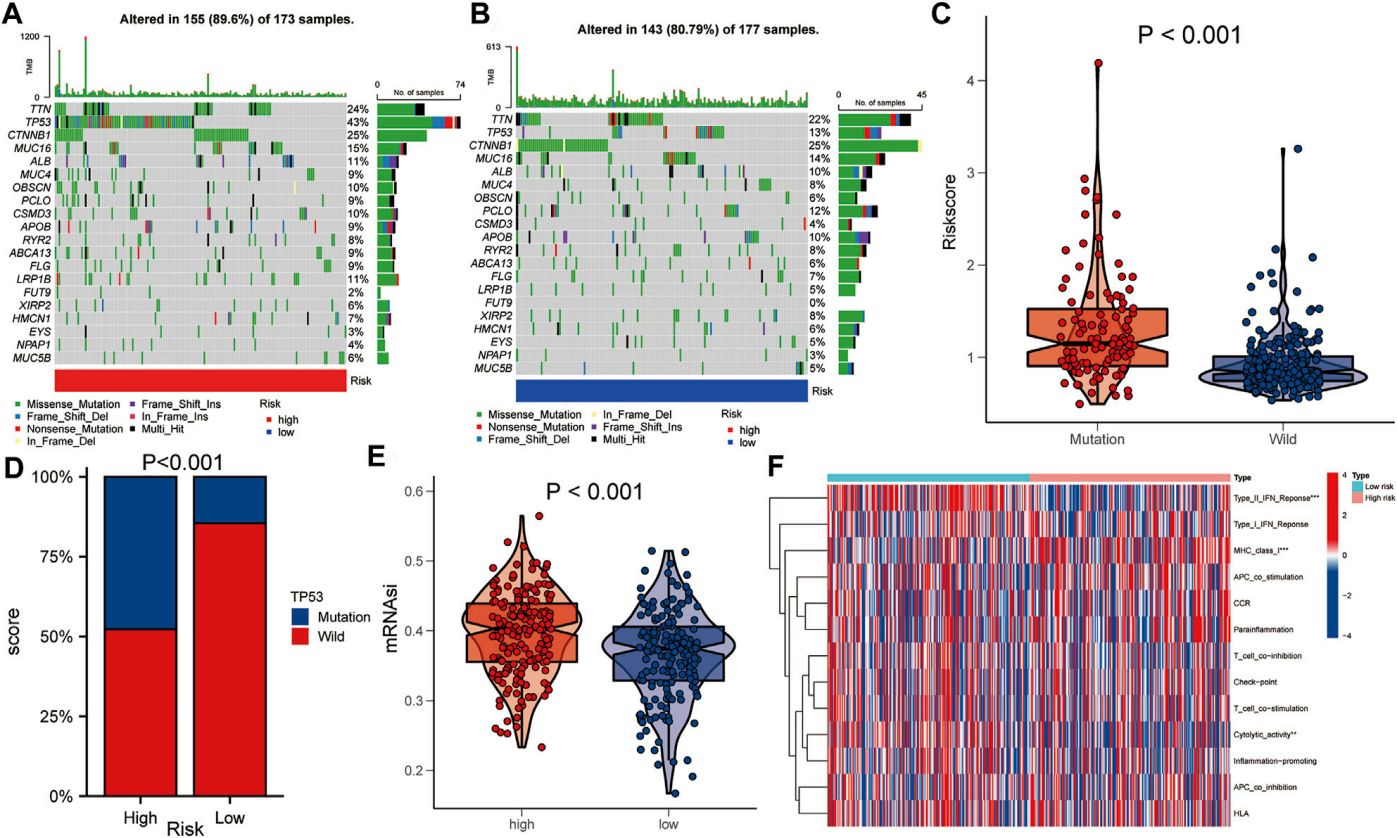
Comparison of gene mutations between the high- and low-risk groups. **(A,B)** Comparison of gene mutation rate between high- and low-risk group. **(C)** Comparison of risk score between TP53 mutation group and TP53 wild group. **(D)** Comparison of TP53 mutation ratio between high- and low-risk group. **(E)** Comparison of mRNAsi between high- and low-risk group. **(F)** Heat map showed the difference in immune function between the high- and low-risk group. Asterisks indicate statistical significance at: *: *p* < 0.05; **: *p* < 0.01, and ***: *p* < 0.001.

The gene mutation data of HCC patients in TCGA was downloaded to compare the gene mutation status between the high- and the low-risk groups. The results showed that there were some differences in gene mutation frequency between the two groups. TP53 gene mutation status between the two groups was significantly different (Figures 6A,B). The risk score of the TP53 mutant group was higher than that of the TP53 wild group (*p* < 0.001, Figure 6C). TP53 mutation rate of the high-risk group was higher than that of the low-risk group (*p* < 0.001, Figure 6D). In addition, the mRNAsi of the high-risk group was higher than that of the low-risk group (*p* < 0.001, Figure 6E). There were statistically significant differences between the high- and low-risk groups in immune function of Type IL IFN Reponse, MHC class I and Cytolytic activity (*p* < 0.01, Figure 6F).

### Correlations Between Risk Score and Tumor Progression in HCC Patients

**FIGURE 7:**
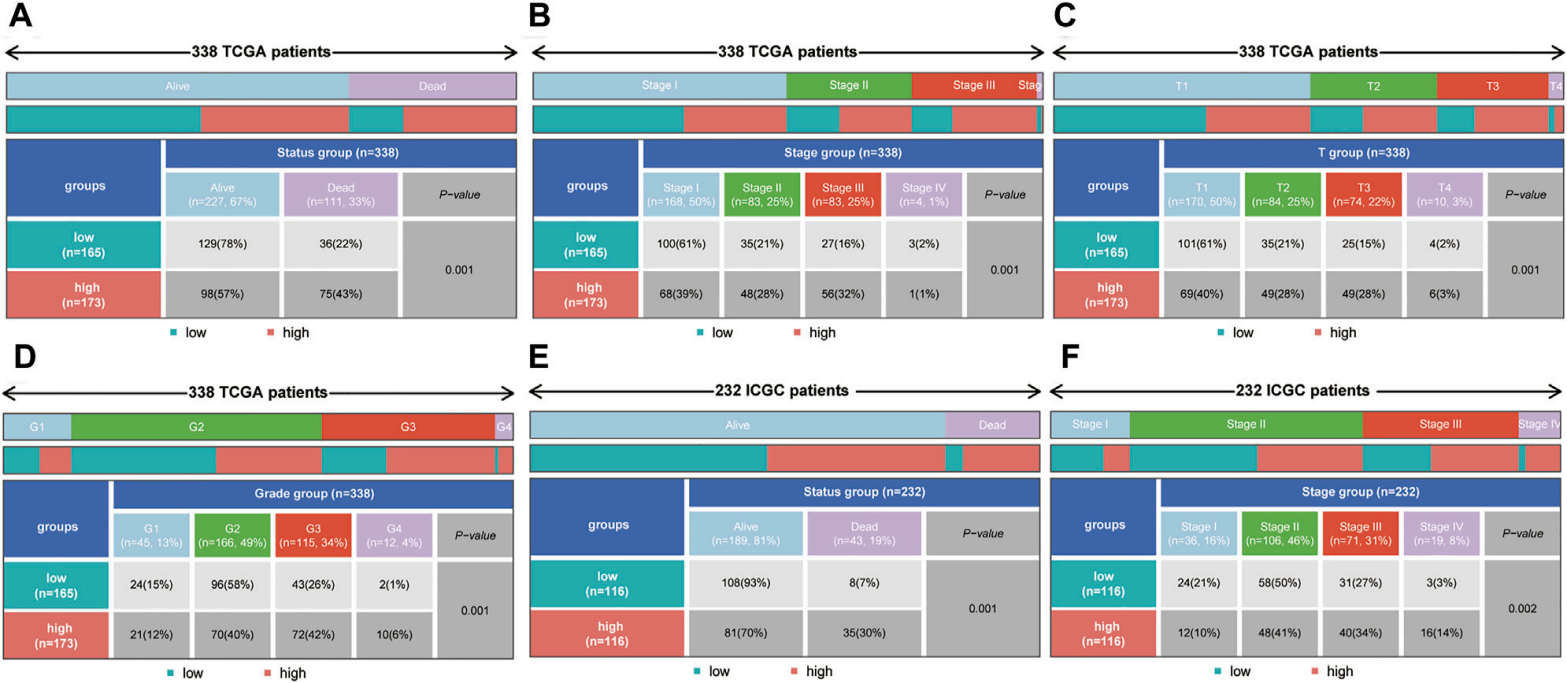
The correlation between the risk score and mortality (or pathological stage) in the training set and validation set. **(A–D)** The high-risk group had higher mortality, more advanced pathological stage, a higher T stage and a higher tumor grade in the training set. **(E,F)** The high-risk group had higher mortality and more advanced pathological stage in the validation set.

To explore the correlations between the risk score and tumor progression, the mortality and pathological stage of the high- and low-risk groups were compared. The results suggested that in the TCGA dataset, the mortality of the high-risk group was higher than that of the low-risk group (*p* = 0.001, Figure 7A). Meanwhile, the proportions of patients with advanced pathological stages (or pathological grades) in the high-risk group were higher than those of the low-risk group (*p* = 0.001, Figures 7B–D). In the ICGC dataset, the mortality rate of the high-risk group was also higher than that of the low-risk group (*p* = 0.001, Figure 7E). At the same time, the proportions of patients with advanced pathological stages (or pathological grades) in the high-risk group were also higher than those of the low-risk group (*p* = 0.002, Figure 7F).

### Risk Score Was an Indicator of Poor Prognosis in the Subgroups Divided by Various Clinicopathological Characteristics

**FIGURE 8:**
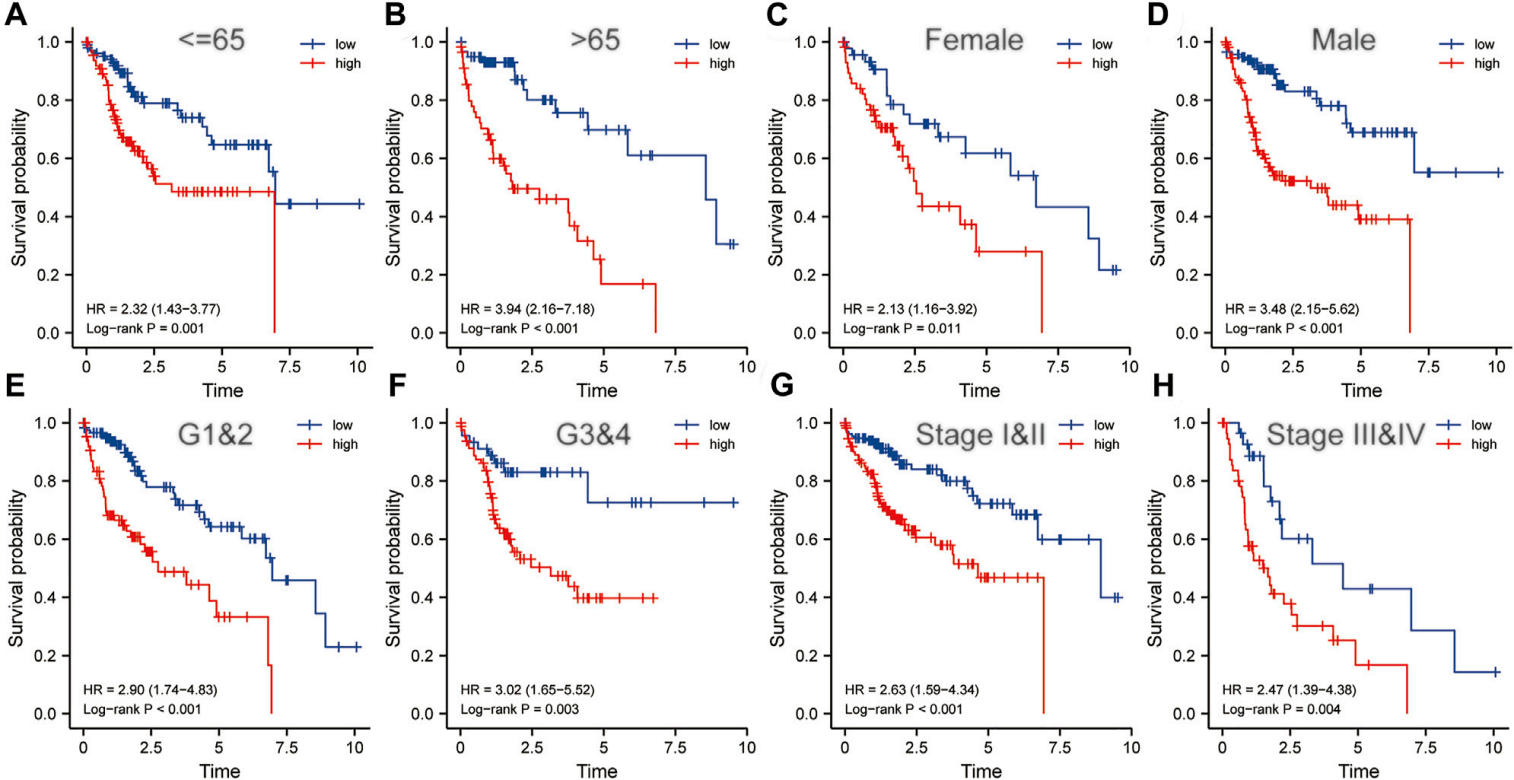
The risk score was an important indicator of poor prognosis in each subgroup divided by clinicopathological characteristics. In various subgroups divided by clinicopathological characteristics including age **(A,B)**, gender **(C,D)**, grade **(E,F)** and pathological stage **(G,H)**, the prognosis of the high-risk groups was poorer than that of the low-risk groups. HR, risk ratio.

Clinicopathological characteristics including age, gender, grade, and pathological stage were used to divide multiple subgroups. As shown in Figures 8A–H, risk scores based on five-gene markers can distinguish high-risk patients with poor prognosis in these subgroups (*p* < 0.001).

### Enrichment Analysis Based on the Risk Score

**FIGURE 9:**
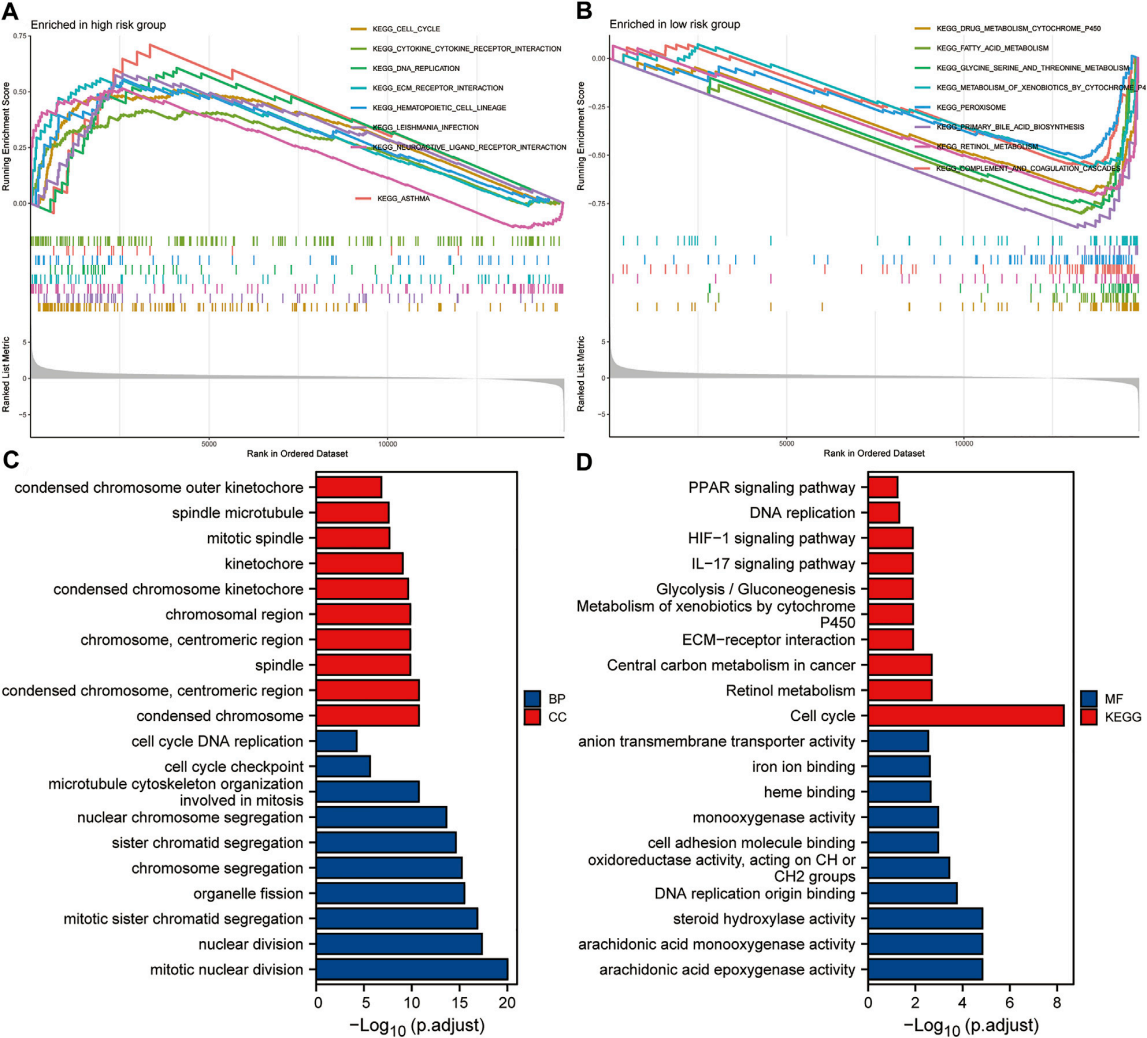
Enrichment analysis based on the risk score. **(A,B)** GSEA analysis of high- and low-risk groups. **(C,D)** GO and KEGG analysis of DEGs between high- and low-risk groups.

Taking the median of the risk scores of all HCC patients from the TCGA dataset as the cut-off value, we divided these samples into high- and low-risk groups. GSEA analysis was conducted to identify the significant enrichment pathways of the high and low risk groups, respectively. Significantly enriched pathways in the high-risk group included cell cycle, cytokine-cytokine receptor interaction, DNA replication, ECM receptor interaction, and hematopoietic cell lineage (Figure 9A). And significantly enriched pathways in the low-risk group included drug metabolism cytochrome p450, fatty acid metabolism, glycine serine and threonine metabolism, metabolism of xenobiotics by cytochrome p450 and peroxisome (Figure 9B). Subsequently, we performed GO and KEGG analysis on DEGs between the high and low risk groups. The results suggested that significantly enriched BP included chromosome segregation, organelle fission, mitotic sister chromatid segregation, nuclear division and mitotic nuclear division; Significantly enriched CC included chromosomal region, chromosome, centromeric region, spindle, condensed chromosome, centromeric region, and condensed chromosome (Figure 9C); Significantly enriched MF includes oxidoreductase activity, acting on CH or CH2 groups, DNA replication origin binding, steroid hydroxylase activity, arachidonic acid monooxygenase activity, and arachidonic acid epoxygenase activity. Besides, significantly enriched KEGG pathways included metabolism of xenobiotics by cytochrome P450, ECM-receptor interaction, central carbon metabolism in cancer, retinol metabolism, and cell cycle (Figure 9D). These results suggested that the five-gene signature may play an important role in tumorigenesis and development.

### Prediction of Immunotherapy Based on Risk Score

**FIGURE 10:**
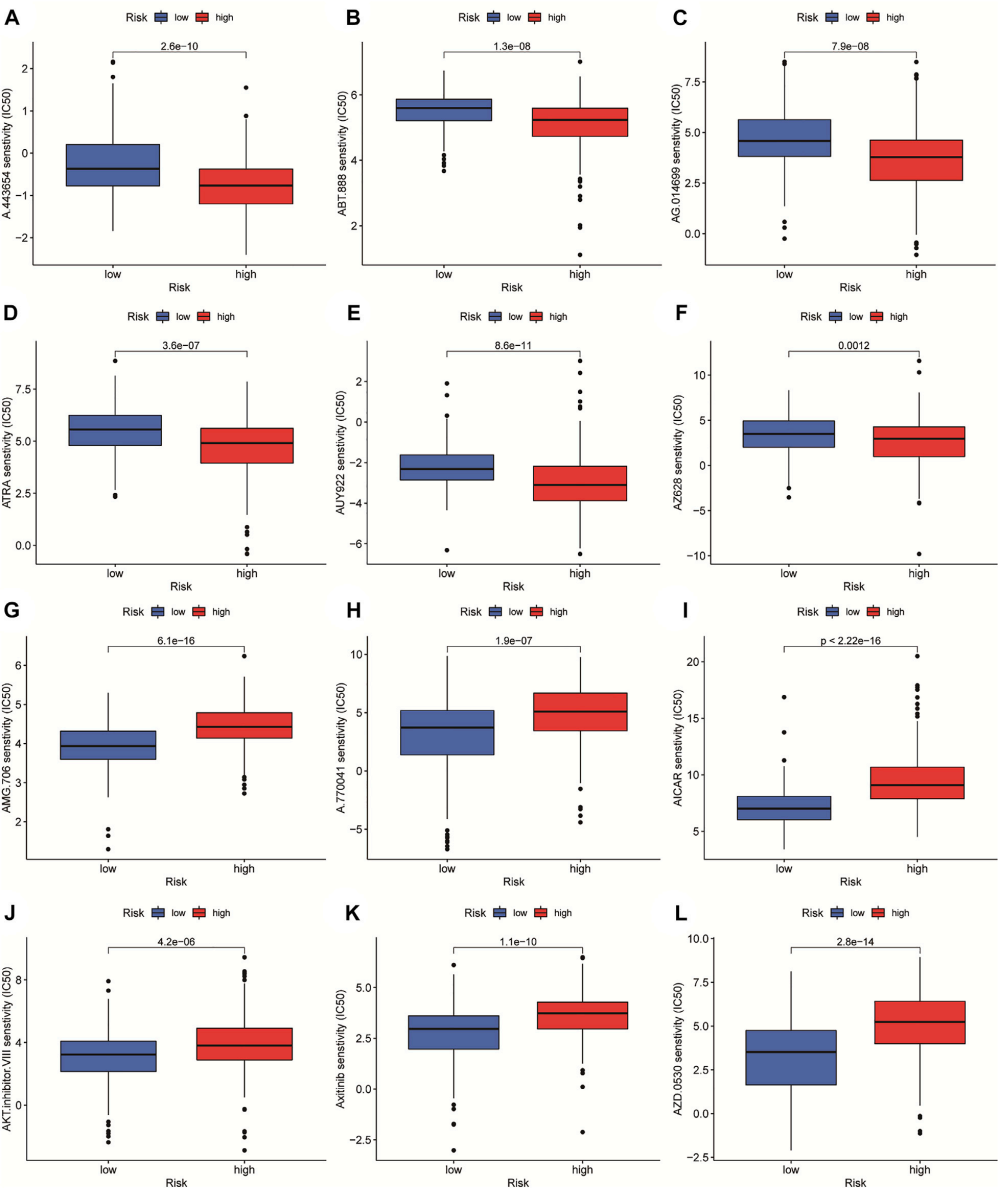
The investigation of tumor immunotherapy. The drugs prediction of risk groups.

In order to select appropriate checkpoint inhibitors for HCC patients, we performed immunotherapy predictions based on risk scores. The results showed that the high-risk group had lower IC50s for six immunotherapy drugs including A-443654, ABT-888, AG-014699, ATRA, AUY-922, and AZ-628, while had higher IC50s for six kinds of immunotherapy drugs including AMG-706, A-770041, AICAR, AKT inhibitor VIII, Axitinib, and AZD-0530 (Figure 10).

### Drug Sensitivity Analysis of Five Hub Genes

To explore the potential correlations between the expressions of five key genes and drug sensitivity, we conducted drug sensitivity analysis using the CellMiner™ database. The results showed that FARSB expression was positively correlated with the drug sensitivity of Hydroxyurea (Supplementary Figure S1A), Vorinostat (Supplementary Figure S1E), Nelarabine (Supplementary Figure S1G), and Lomustine (Supplementary Figure S1P), while negatively correlated with the drug sensitivity of JNJ-42756493 (Supplementary Figure S1O). DHX37 expression was positively correlated with the drug sensitivity of Raltitrexed (Supplementary Figure S1B), Cytarabine (Supplementary Figure S1D), Cisplatin (Supplementary Figure S1F), Thiotepa (Supplementary Figure S1H), and Triethylenemelamine (Supplementary Figure S1N). YARS expression was positively correlated with the drug sensitivity of Axitinib (Supplementary Figure S1C), Fluphenazine (Supplementary Figure S1K), and Megestrol acetate (Supplementary Figure S1M). NOP58 expression was positively correlated with the drug sensitivity of Vorinostat (Supplementary Figure S1I) and 6-Thioguanine (Supplementary Figure S1J). The expression of CCT4 was positively correlated with the drug sensitivity of Nelarabine (Supplementary Figure S1L).

### Constructing a Nomogram to Predict OS in HCC Patients

**FIGURE 11:**
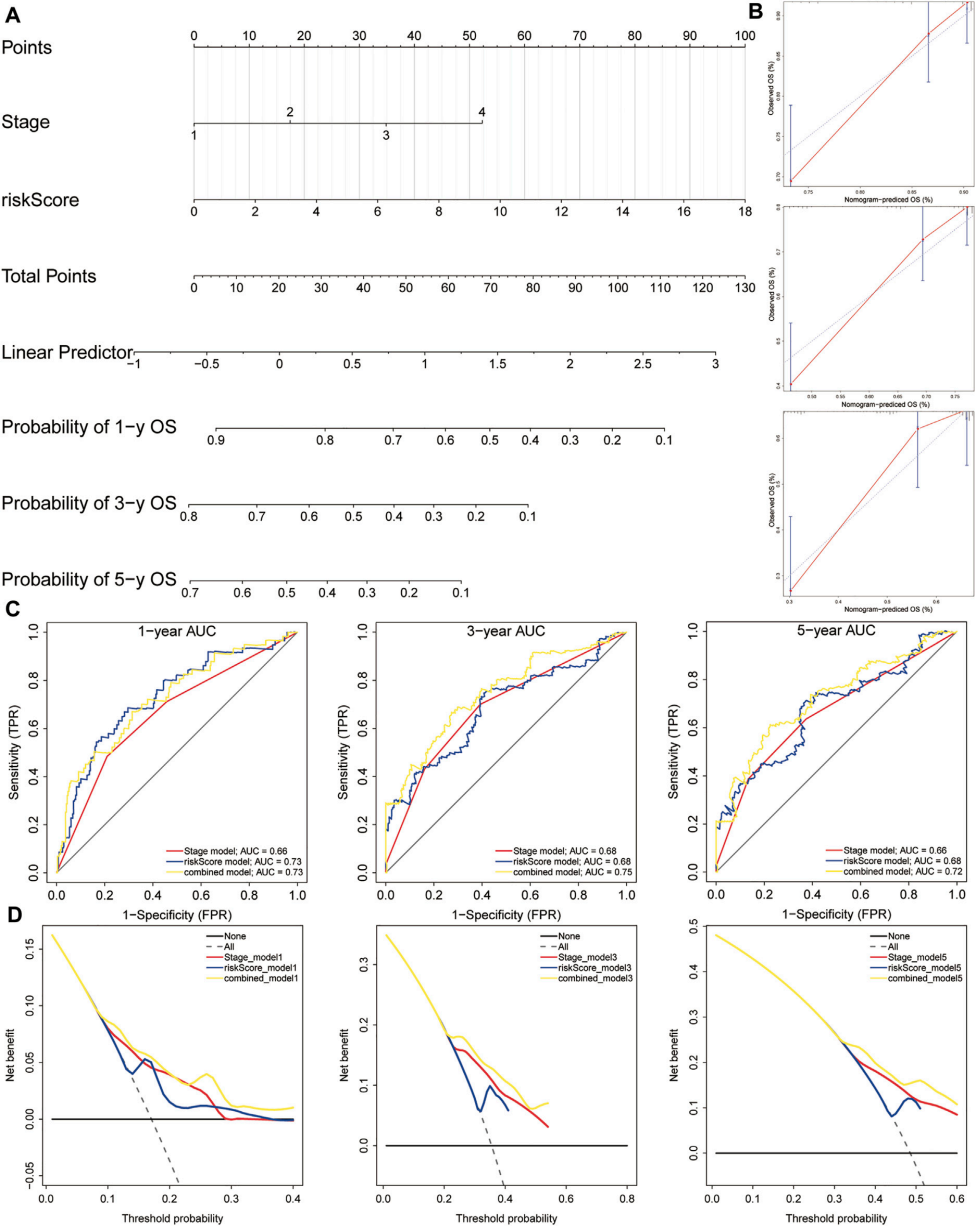
A nomogram used for survival prediction. **(A)** A nomogram combining the five-gene signature and clinical pathological stage. **(B)** The calibration chart showed that the predicted 1-, 3-, and 5-year survival probabilities were basically consistent with actual observations. **(C)** The t-ROC analysis showed that the nomogram had good survival prediction power. **(D)** DCA curve visually evaluated the clinical benefit of the nomogram and the scope of application of the clinical benefit obtained by the model. The calculated net benefit (*Y* axis) was plotted against the threshold probabilities of patients with 1-, 3-, and 5-year survival on the *X* axis. The gray dotted line represents the hypothesis that all patients have 1-year, 3-year, and 5-year survival. The solid black line represents the hypothesis that no patient has a 1-year, 3-year, or 5-year survival period. t-ROC, time-dependent receiver operating characteristics; DCA, decision curve analysis.

In order to establish a clinically applicable method for predicting the OS of HCC patients, we constructed a nomogram combining risk score and pathological stage (Figure 11A), and then analyzed the accuracy of the model using a calibration curve. The results showed that the 1-year, 3-year, and 5-year survival probabilities predicted by the nomogram were basically consistent with the observed survival probabilities, confirming the reliability of the nomogram (Figure 11B). Meanwhile, t-ROC curve suggested that the nomogram combined with pathological stage and risk score had the largest AUC. The AUCs of 1-, 3-, and 5-year survival predictions were above 0.72, which suggested that compared with the model constructed by a single prognostic factor, the nomogram combining risk scores and pathological stages was a better prognostic model for survival prediction in HCC patients (Figure 11C). In addition, we plotted the calculated net benefit with the threshold probabilities for HCC patients with 1-year, 3-year, and 5-year survival rates. As shown in Figure 11D, the net benefit of the nomogram was better than other models.

## Discussion

In recent years, immunotherapy has become the focus of HCC research. Immune checkpoint inhibitors, including anti-PD-1, anti-PD-L1, and anti-CTLA-4 antibodies, have shown potential therapeutic value in advanced HCC. At present, the anti-PDL1 antibody Atezolizumab combined with the vascular endothelial growth factor neutralizing antibody Avastin is expected to become the standard treatment for HCC. Therefore, HCC checkpoint immunotherapy combined with other systemic or local treatments is considered to be the most promising treatment option for HCC. Currently, there is an urgent need for the identification and validation of predictive biomarkers and the screening of more effective immunotherapy drugs for HCC immunotherapy.

In this study, we focused on constructing a proliferation-related gene signature for patients with HCC. Firstly, the CRISPR Library and the TCGA database were used to screen differentially expressed genes related to the proliferation of HCC cells. Then, univariate COX regression analysis, random forest algorithm and multiple combinations were used to construct a prognostic five-gene signature (FARSB, NOP58, CCT4, DHX37, and YARS). Next, the prognostic value of the five-gene signature was confirmed in both the training set and the validation set. Finally, we combined risk scores and pathological stage to construct a nomogram for clinical practice. Meanwhile, calibration curve, ROC curve and decision curve showed that the nomogram can more accurately predict the ability of OS in HCC patients. In addition, the roles of this five-gene signature in gene mutation, cancer cell stemness and immune functions were explored, respectively. Therefore, this five-gene signature is an independent prognostic predictor of HCC.

Traditional pathological staging is commonly used method for evaluating the prognosis of HCC patients. Alpha-fetoprotein (AFP) is a widely used biomarker to monitor treatment response and improve prognosis. However, the high heterogeneity of HCC increases the difficulty of survival prediction. Recently, some new biomarkers have become effective tools for predicting the prognosis of HCC. For example, CCL14, CBX3/HP1, APEX1, and UBE2C are considered to be prognostic biomarkers for HCC (Wei Z. et al., 2019; Zhong X. et al., 2019; Cao et al., 2020; Gu et al., 2020). In addition, a four-gene signature including PBK, CBX2, CLSPN, and CPEB3, a four-methylated mRNA signature including BRCA1, CAD, CDC20, and RBM8A, a 5-gene lncRNA signature including RP11-325L7.2, DKFZP434L187, RP11-100L22.4, DLX2-AS1, and RP11-104L21.3, as well as many other polygenic gene signatures have been shown to have prognostic value in HCC (Sun et al., 2019; Wang Y. et al., 2019; Yan et al., 2019).

Based on the important role of HCC cell proliferation in tumor progression and its impact on patient prognosis, this work constructed a prognostic gene signature associated with HCC cell proliferation. The results showed that the five-gene signature including FARSB, NOP58, CCT4, DHX37, and YARS was with good prognostic values. Meanwhile, the enrichment analysis showed that the significant enrichment pathways in the high-risk group included cell cycle, cytokine-cytokine receptor interaction, DNA replication, ECM receptor interaction and hematopoietic cell lineage. These results indicated that the five hub genes were involved in the molecular mechanism of proliferation and progression in HCC. Previous studies have shown that FARSB is involved in amino acid metabolism and tRNA aminoacylation, and plays a key role in the progression of gastric cancer (Gao et al., 2021). NOP58 is involved in the transport of mature mRNA and protein metabolism that do not depend on SLBP. NOP58 is not only negatively related to the OS of HCC patients, but may also be closely related to the recurrence of lung adenocarcinoma (Shen et al., 2021; Wang et al., 2021). CCT4 is involved in protein metabolism and is significantly related to HCC cell growth and prognosis (Li F. et al., 2021; Li W. et al., 2021). In addition, downregulation of CCT4 can significantly inhibit the migration of lung adenocarcinoma cells (Tano et al., 2010). DHX37 is an RNA helicase, which is significantly upregulated in 17 kinds of tumors (Huang et al., 2021). DHX37 could affect the prognosis of patients with HCC or lung adenocarcinoma by immune infiltration, and can be used as a prognostic biomarker for HCC and lung adenocarcinoma (Xu et al., 2020; Chen et al., 2022). Moreover, DHX37 acts as a function regulator of CD8 T cells (Dong et al., 2019). YARS1 is involved in tRNA aminoacylation and gene expression. There are no reports on the role of YARS1 in HCC for now.

It is worth mentioning that the gene signatures constructed by different methods may have different applications. For example, a four-gene metabolic signature for HCC can reflect the disorder of the metabolic microenvironment, thereby providing potential biomarkers for the metabolic treatment and treatment response prediction of HCC (Liu et al., 2020). A ferroptosis-related gene signature can be used to predict the prognosis of HCC patients (Liang et al., 2020). An immune-related lncRNA signature has the potential to measure the response to ICB immunotherapy and guide the choice of HCC immunotherapy (Zhang Y. et al., 2020). An immune-related gene signature can predict the response of HCC patients to immunotherapy (Dai et al., 2021). DNA methylation is an important regulator of gene transcription in the etiology and pathogenesis of HCC. Two HCC prognostic signatures related to DNA repair have recently been reported to help explore molecular mechanisms related to DNA repair (Li N. et al., 2019; Li G. X. et al., 2019). A gene signature related to glycolysis could help to analyze the role of glycolysis in HCC (Jiang et al., 2019). In addition, the tumor microenvironment plays an important role in the progression, recurrence and metastasis of HCC. A gene signature based on the HCC microenvironment helps to explore the role of the tumor microenvironment in HCC (Zhang F.-P. et al., 2020).

This study has some limitations. Although this study used the method of mutual verification between two independent datasets to verify the prognostic significance of the five-gene signature. However, *in vitro* experiments are still an important step to further confirm the prognostic value of this gene signature. In addition, this is a retrospective study, so it is necessary to verify the robustness of this five-gene signature in a prospective study in the future.

## Conclusion

In summary, the study identified a new prognostic gene signature based on proliferation-related genes (FARSB, NOP58, CCT4, DHX37, and YARS). Besides, a nomogram based on the five-gene signature was constructed for clinical practice. The five-gene signature can be used for survival prediction and risk stratification for HCC patients. Moreover, potential drugs targeting this gene signature deserve further attention in the treatment of HCC.

## Data Availability

The datasets presented in this study can be found in online repositories. The names of the repository/repositories and accession number(s) can be found in the article/Supplementary Material.
