# Supplementary material for: Machine Learning Screens Potential Drugs Targeting a Prognostic Gene Signature Associated With Proliferation in Hepatocellular Carcinoma
Source: Front Genet. 2022 Jun 28;13:900380. doi: 10.3389/fgene.2022.900380 (PMC9273781; doi:10.3389/fgene.2022.900380)
Supplement: Supplementary file 1 [file DataSheet1.docx]

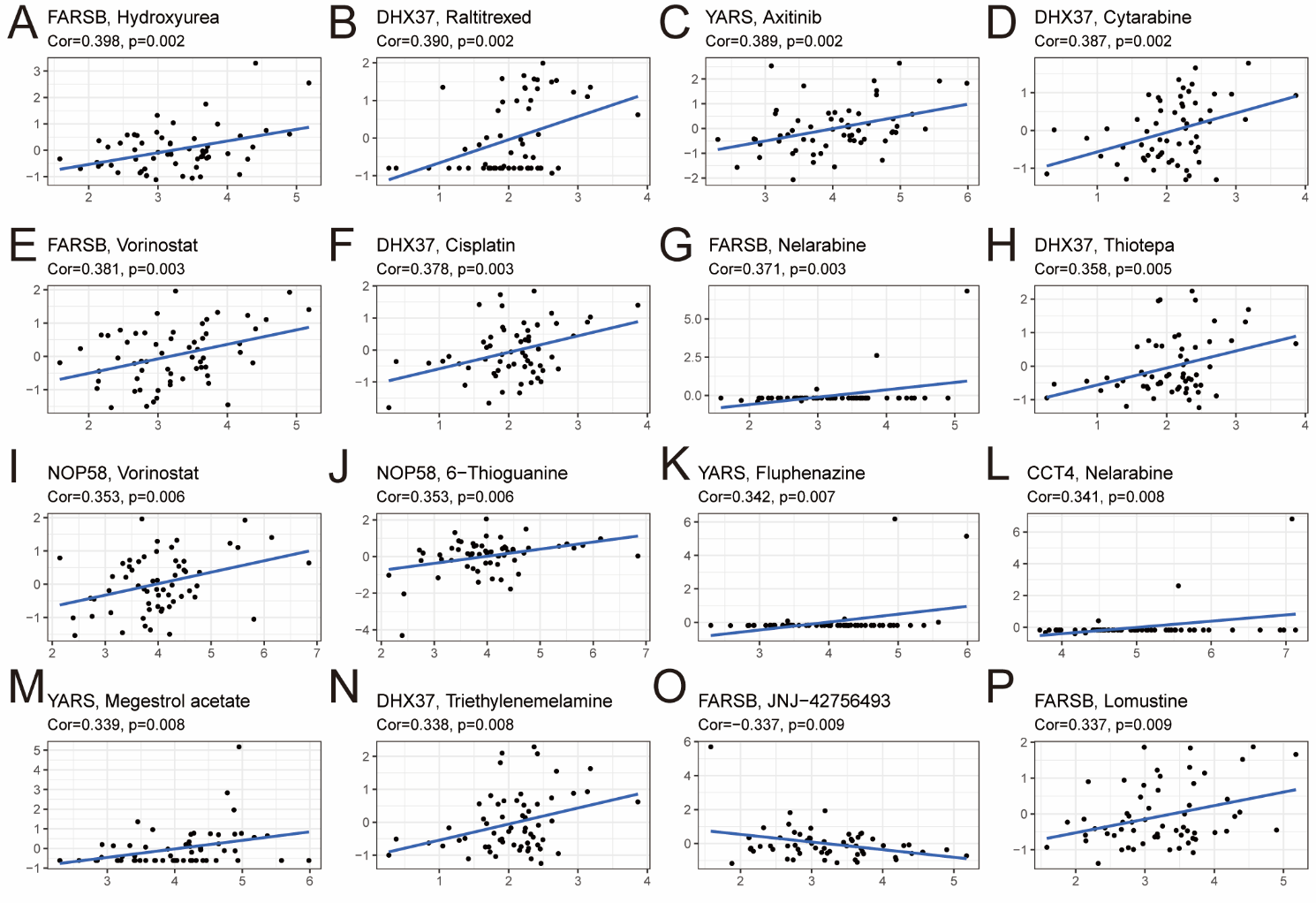


**Figure S1. Drug sensitivity analysis of five hub genes.** FARSB expression was positively correlated with the drug sensitivity of Hydroxyurea **(A)**, Vorinostat **(E)**, Nelarabine **(G)**, and Lomustine **(P)**; FARSB expression was negatively correlated with drug sensitivity of JNJ-42756493 **(O)**; DHX37 expression is positively correlated with drug sensitivity of Raltitrexed **(B)**, Cytarabine **(D)**, Cisplatin **(F)**, Thiotepa **(H)** and Triethylenemelamine **(N)**; YARS expression was positively correlated with drugs sensitivity of Axitinib **(C)**, Fluphenazine **(K)** and Megestrol acetate **(M)**; NOP58 expression was positively correlated with drug sensitivity of Vorinostat **(I)** and 6-Thioguanine **(J)**; CCT4 expression was positively correlated with drug sensitivity of Nelarabine **(L)**. Cor, correlation coefficient.
